# Adherence to Chinese Dietary Guidelines Is Associated with Better Bone Status in School-Aged Children and Adolescents

**DOI:** 10.3390/nu18111812

**Published:** 2026-06-04

**Authors:** Shiyi Ouyang, Ailing Chen, Yan Li, Wenlong Lu, Jiali Cai, Jiaren Liu, Zhuang Ma, Yuhan Tang, Ping Yao, Ting Xiong, Jingfan Xiong, Yanyan Li, Yuanjue Wu

**Affiliations:** 1 Department of Nutrition and Food Hygiene, School of Public Health, Guangzhou Medical University, Guangzhou 511436, China; 2 Shenzhen Center for Chronic Disease Control, Shenzhen 518020, China; 3 Department of Nutrition and Food Hygiene, School of Public Health, Tongji Medical College, Huazhong University of Science and Technology, Wuhan 430030, China

**Keywords:** Chinese dietary guidelines index, dietary pattern, bone health, quantitative ultrasound, children and adolescents

## Abstract

**Background/Objectives:** Diet plays a crucial role in bone health; however, most studies have focused on individual nutrients or foods. The relationship between overall dietary quality and bone health in children and adolescents remains unclear. This study aimed to assess the association between diet quality scores and bone health in a pediatric population. **Methods:** A total of 3299 students aged 9–17 participated in this study. Dietary quality was assessed using the Chinese dietary guidelines index for Children and Adolescents (CDGI-Cs). Bone health was evaluated through calcaneal quantitative ultrasound (QUS), utilizing the speed of sound (SOS) as the key indicator. Generalized linear models and binary logistic models were used to analyze the association between CDGI-C scores and bone health outcomes (SOS Z scores and the risk of low SOS), respectively. **Results:** After full adjustment, CDGI-C scores showed a significant positive association with SOS. A 10-point increase in CDGI-C scores was associated with a 0.08-unit increase in SOS Z score (95% CI: 0.04, 0.11) and a 15% reduction in the risk of low SOS (OR = 0.85, 95% CI: 0.76, 0.94). Compared to participants in the lowest quartile of CDGI-C scores, those in the highest quartile exhibited significantly higher SOS Z scores (β = 0.18, 95% CI: 0.08, 0.29, *p* for trend < 0.001), and a 29% lower risk of low SOS (OR = 0.71, 95% CI: 0.50, 1.00, *p* for trend = 0.050). In dietary item analysis, higher intake of dairy and dairy products (β = 0.02, 95% CI: 0.00, 0.03) and seafood (β = 0.01, 95% CI: 0.00, 0.03) remained positively associated with SOS Z scores. **Conclusions:** Higher diet quality, as measured by CDGI-C, was significantly associated with better bone health in children and adolescents. Dairy, dairy products, and seafood emerged as key dietary components contributing to this positive association.

## 1. Introduction

In recent years, numerous studies have demonstrated that bone health is essential for maintaining lifelong skeletal structure and function [1]. Childhood and adolescence represent critical periods for bone mass accumulation and optimal skeletal development. By the end of puberty, adolescents accumulate at least 90% of their adult peak bone mass [2,3], which also correlates with their future risk of osteoporotic fractures [2,3]. Loss of bone mass and disruption of the microarchitecture of the bones are the main symptoms of osteoporosis (OP), a systemic bone disease [4,5], which has emerged as an increasingly severe public health challenge against the backdrop of an accelerating aging population [6,7,8]. Therefore, investigating the determinants of bone health during childhood is of critical importance.

Among the various modifiable factors impacting bone health, diet is essential in shaping and preserving the integrity of bones [9]. Over the years, research on diet and bone health has largely focused on the effect of a single nutrient or food (e.g., calcium, vitamin D, and dairy products) [10,11,12]. However, daily diets represent complicated mixtures of diverse foods and nutrients, and also, a single component cannot adequately account for the synergistic or antagonistic effects that dietary patterns may have on health [13,14]. By measuring a person’s diet corresponding to national dietary guidelines, dietary quality indices offer an effective method to assess overall dietary quality [15]. Internationally, such indexes (e.g., the Healthy Eating Index, HEI) have been demonstrated to correspond with better bone health in both adult and adolescent populations [9,16]. However, as Kindler et al. emphasized, most dietary-bone studies in children still employ data-driven dietary pattern analysis rather than a priori dietary quality index that corresponds to dietary guidelines [9]. In recent years, the Chinese dietary guidelines index for Children and Adolescents (CDGI-Cs) has been established based on the Dietary Guidelines for Chinese Residents (2016) [17]. This index encompasses 14 dietary components, enabling a comprehensive assessment of dietary quality.

However, the application of CDGI-C in bone health research is still in its early stages and has several limitations, such as the inability to cover all dietary indicators of CDGI-C due to data limitations. For example, a Chinese study that applied the CDGI-C dietary pattern lacked information on dark green vegetables, salt, oil, and sugar intake, possibly because the quantification methods for these variables are more challenging than those for other dietary indicators [18]. Furthermore, there is very little evidence linking CDGI-C components to bone health.

While the CDGI-C serves as a reliable tool for assessing overall dietary quality in children and adolescents, its relationship with bone health assessed by calcaneal quantitative ultrasound (QUS) remains unclear, both as a continuous score and the independent contributions of dietary components. To fill this knowledge gap, this study investigated the relationship between CDGI (2021)-C scores and various dietary components with calcaneal speed of sound (SOS) measured by QUS among children and adolescents. We predict that higher CDGI-C will be associated with higher SOS levels, indicating better bone health status.

## 2. Materials and Methods

### 2.1. Study Population

This study utilized data from the Evaluation and Monitoring on School-based Nutrition and Growth in Shenzhen (EMSNGS) (Chinese clinical trial registry: ChiCTR2100051722; https://www.chictr.org.cn (accessed 20 December 2023)). It is a continuous cohort study of children and adolescents established and managed by the Shenzhen Center for Chronic Disease Control and Prevention. The baseline survey was completed in 2021 using a multistage stratified cluster random sampling approach [19]. The primary goal of this cohort is to assess and track the nutritional health, growth, and development of 6–18-year-old children and adolescents in Shenzhen. Full information on the cohort protocol and objectives was provided to all study participants and their legal guardians. Written informed consent was obtained from all participants and their legal guardians. All subject data were collected by trained professionals, ensuring high data quality and completeness. The Ethics Review Committee of the Shenzhen Center for Chronic Disease Control (No. SZCCC-2021-037-01-PJ, 25 April 2021) approved this study.

**Figure 1:**
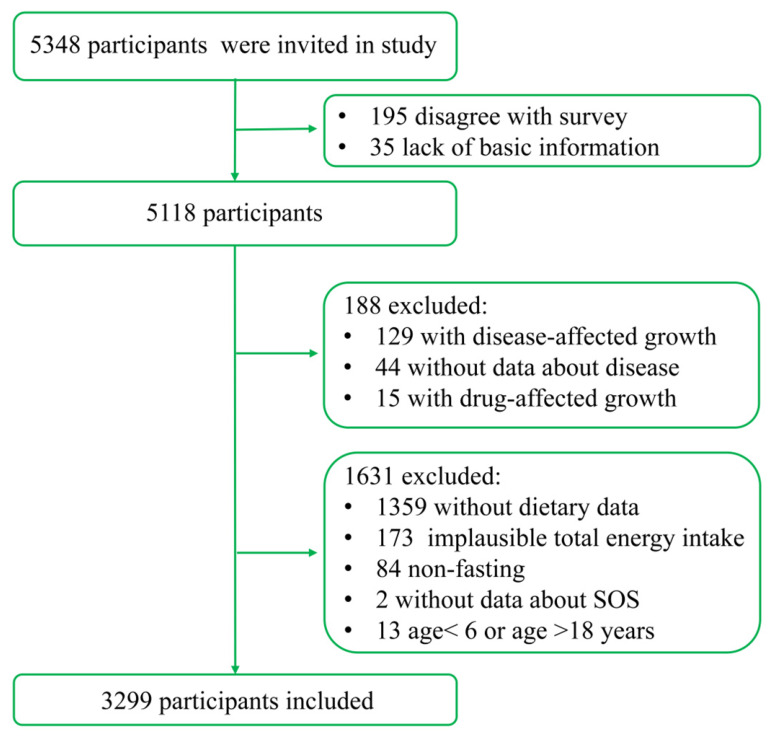
Flow chart of the participants in this study.

A total of 5348 participants were initially enrolled in this study. Of these, 35 participants were excluded because they lacked the basic information, and 195 participants were excluded for not providing informed consent. Participants lacking information on SOS (*n* = 2) and dietary nutrient intake information (including children in grades 1–3 for whom detailed dietary data were not collected, *n* = 1363) were excluded, and those with unreasonable total energy intake (*n* = 173) were also excluded. Additionally, exclusion criteria included defects from birth, genetic defects in development, hormonal disorders, chronic liver and kidney disease, autoimmune disorders, and blood or tumor illnesses (*n* = 129), as well as participants without knowledge of these conditions (*n* = 44). Furthermore, we removed 85 participants because they were fasting, and those who were not between the ages of 6 and 18 (*n* = 23) were excluded. Ultimately, 3299 subjects were contained in the study analysis. A comprehensive flowchart was presented in Figure 1.

### 2.2. Assessment of CDGI-C

A validated Food Frequency Questionnaire (FFQ) developed for Chinese children and adolescents was used in this survey after the addition of questions about local diets [20,21]. It covered 12 food groups, including cereals and tubers, legumes, vegetables and fruits, dairy products, mushrooms and algae, meat, beverages and drinking water, and nuts and snacks, totaling 61 food items. Trained nutritionists collected data through face-to-face interviews, using standardized food images to help participants understand portion sizes and recall intake frequency (daily, weekly, monthly, or rarely/never). Experienced nutritionists provided guidance to participants, who estimated their food intake using food models representing standard portion sizes and food pictures depicting different portion sizes for all food items. Participants were asked to report their usual frequency of consuming each food item and the average serving size over the past month. For younger participants, parental assistance was provided during the interview to enhance recall accuracy.

We employed the CDGI (2021)-C to assess dietary quality. The CDGI (2021)-C was developed based on the recommended food intake for children and adolescents in the Dietary Guidelines for Chinese Residents (2016) and the balanced diet pyramid, using an equal-weight continuous scoring method, with a score range from 0 to 110 points. It does not include evaluations of physical activity or water intake. It offers a complete assessment of dietary quality among Chinese children and adolescents and has been employed in existing studies [18]. The assessment system comprises 14 specific dietary indicators. Firstly, adequacy components include vegetables, dark green vegetables, dairy and dairy products, fruits, nuts, and beans. These foods should be consumed in sufficient quantities to meet nutritional needs. Secondly, moderation components include carbohydrate (CHO) rate, grain and legume, seafood, meat and egg, which should be consumed in moderation to maintain dietary balance. No upper limit is set for maximum recommended seafood intake because insufficient seafood intake is common among children and adolescents, so it is scored as an adequate intake category. Then, restriction components include oil, salt, and drinks, which should be consumed in limited quantities to avoid health issues from excessive intake. The CDGI (2021)-C score quartile was used to categorize all participants into low- to high-quartile groups (Q1, Q2, Q3, and Q4). Table S1 provides specifics on the calculation method.

### 2.3. Assessment of Bone Health

Bone health was measured by calcaneal QUS, using the CM-200 densitometer (Furuno Electric, Nishinomiya City, Japan). Recently, QUS has emerged as a preferred tool for bone health assessment in children and adolescents in bone health assessments, owing to its non-invasive and radiation-free characteristics [22]. Compared to Dual-Energy X-ray Absorptiometry (DXA), this method is more economical, provides portable equipment, and prevents radiation exposure [23]. Two commonly generated QUS parameters are SOS and broadband ultrasound attenuation (BUA). Among these, SOS represents the division of the sound waves’ transmission time by the length of the body part under study, expressed in m/s [22]. Notably, the speed of sound through bone varies with the density and elastic modulus of the tissue, making it a direct indicator of bone mineral density and structural integrity. SOS is a key indicator providing crucial information about bone mass and structure [22], with higher values typically indicating greater bone tissue density. Numerous studies have also confirmed the close association between SOS and both bone tissue density and fracture risk [24,25,26]. The standard deviation of a subject’s SOS in relation to the mean SOS of a reference population of the same age and gender is represented by the SOS Z score, which was used to describe the degree of deviation in children’s and adolescents’ SOS. The SOS Z score is calculated as [(SOS—Mean SOS for same-race, -sex, -age peers)/Standard deviation of SOS for same-race, -sex, -age peers]. Low SOS is indicated by an SOS Z score < −1.0 and an SOS Z score ≥ −1.0 is regarded as normal.

### 2.4. Other Covariates

Participants’ lifestyle, health, sociodemographic, and economic characteristics were acquired through thorough questionnaire interviews. Additionally, factors needing anthropometric measurements were precisely measured by specialists using defined methods. The health status question covered the subject’s personal medical history, present illnesses, and medication use, including whether they had growth issues or had used drugs impacting growth. Individuals’ weight status is classified as underweight, normal weight, overweight, or obese according to age- and gender-specific body mass index (BMI), which was calculated as weight divided by the square of height. The following are definitions of the particular evaluation criteria: obesity (BMI ≥ 95th percentile), overweight (85th ≤ BMI < 95th percentile), and underweight (BMI ≤ 5th percentile) [27,28]. The following three categories are used to classify household income: <Chinese yuan (CNY) 120,000 per year, CNY 120,000 to CNY 250,000 per year, and ≥CNY 250,000 per year. Parental education level was categorized into the following four groups: ≤9 years, 9–12 years, 12–15 years, and >15 years. Smoking status was classified as never smoked and smoking. There were the following three categories for drinking status: never, less than one standard drink per month, and one standard drink or more per month. Daily moderate-to-vigorous physical activity (MVPA) levels were categorized as <0.5 h/day, 0.5–0.9 h/day, 1.0–2.9 h/day, and ≥3 h/day. Pubertal development stages were staged by pediatrician from department of endocrinology according to the Tanner staging system (ranging from Tanner stage I–V) and further categorized as pre-puberty (Tanner stage I), mid-puberty (Tanner stages II–III), and post-puberty (Tanner stage IV and above) [29,30,31].

### 2.5. Statistical Analyses

First, all continuous variables were tested for normality by using the Kolmogorov–Smirnov test. The mean ± standard deviation (Mean ± SD) was used to report variables with a normal distribution. For non-normally distributed variables, they were described using medians and interquartile ranges [Median (IQR)]. Categorical variables were described as frequency (n) and percentage (%). Analysis of variance (ANOVA) was employed to analyze continuous variables with normality assumptions, and non-normally distributed continuous variables were assessed with the Kruskal–Wallis rank test. Categorical variables were evaluated using the chi-square test.

Restricted cubic splines (RCSs) with three knots were used to evaluate the dose–response relationships between CDGI-C and SOS Z scores, the risk of low SOS. The association between CDGI-C and SOS Z scores was examined using generalized linear regression models, with results expressed as regression coefficients (β) with 95% confidence intervals (CIs). Additionally, we employed binary logistic regression models to examine the association between CDGI-C scores and the risk of low SOS, with results expressed as odds ratios (ORs) with 95% confidence intervals (CIs). To control for confounding factors, we use the following four regression models for each type: Model 1 was an unadjusted crude model; Model 2 adjusted for age, sex, and weight status; Model 3 further adjusted for parental education, household income, MVPA, smoking status, drinking status, daily energy intake and stage of puberty; and Model 4 additionally adjusted for vitamin D and calcium supplement. Similarly, regression models were established to examine the association between the 14 items of CDGI-C and SOS, where Models 1–4 were consistent with the previous approach, and Model 5 further adjusted for 13 other dietary items of CDGI-C. Subsequently, we explored the association between CDGI-C and SOS across various subgroups, which included gender (male or female), age (9–10, 11–13 or 14–17 years), stage of MVPA (<0.5, 0.5–1, and ≥1 h per day), and weight status (underweight, normal, overweight or obese). The log-likelihood ratio test was used to test the *p* values for interaction.

The statistical analysis was performed using R software version 4.5.1. A two-tailed test was applied with a significance level of α = 0.05 and *p*-value < 0.05, which were considered statistically significant. To control for Type I error inflation due to multiple comparisons, we applied the Benjamini–Hochberg false discovery rate (FDR) correction to all *p*-values from the Kruskal–Wallis tests. Q-values (FDR-adjusted *p*-values) < 0.05 were considered statistically significant.

## 3. Results

### 3.1. Baseline Characteristics

This study included 3299 participants, with a mean CDGI-C score of 64.35 and a mode of 66.38. This indicates that the overall dietary quality of participants was moderate, with scores ranging from 19.24 to 98.41. Participants were classified into low- to high-quartile groups (Q1–Q4) according to CDGI-C (cutoff points were 56.90, 64.84 and 72.48). Table 1 presented the basic characteristics and lifestyle factors of the 3299 participants based on CDGI-C quartiles. Also, the baseline scores of 14 dietary items in the CDGI-C according to CDGI-C quartile were shown in Table 2. Grain and legumes, vegetables, nuts, meat, and eggs had the lowest mode scores among all participants, whereas dark green vegetables, beans, carbohydrate rate, fruit, dairy and dairy products, seafood, salt, oil, and drink had the maximum scores. Furthermore, we observed that the mode for grain and legumes, vegetables, and meat scores was 0 across Q1–Q4 groups, while the mode for nut and eggs scores was 0 in the Q1–Q3 groups. The mode for seafood was 0 in Q1, which was notable, as inadequate seafood intake is more significant with lower scores. This suggests that insufficient intake of grain and legumes, vegetables, and nuts, as well as improper intake of meat and eggs, continues to exist even within groups with relatively higher dietary quality. Notably, for dark green vegetable scores, the median value was 5.00 across all four CDGI-C quartiles, with similar interquartile ranges except for the lower end of Q1. Although the Kruskal–Wallis test yielded a *p*-value < 0.001, this result was largely driven by the large sample size and the presence of many tied values. Therefore, we interpreted this finding with caution, and the descriptive statistics indicated only minimal differences across quartiles. The same pattern was observed for drink scores.

Compared with participants in the lowest quartile of CDGI-C score (Q1), those with higher CDGI-C tended to be younger in age and more likely to be male. Additionally, they were more inclined to have normal weight, as well as not to drink or smoke. Participants with higher CDGI-C scores also tended to spend more time in MVPA than those in the lowest quartile (Q1). Socioeconomic variables were also related to CDGI-C. Higher CDGI-C scores were more common among participants whose parents had higher household incomes and higher educational levels, suggesting a potential positive association between family background and overall diet quality. These participants were reported to be more positively taking vitamin D and calcium supplements.

### 3.2. Association Between CDGI-C and Bone Health

**Figure 2:**
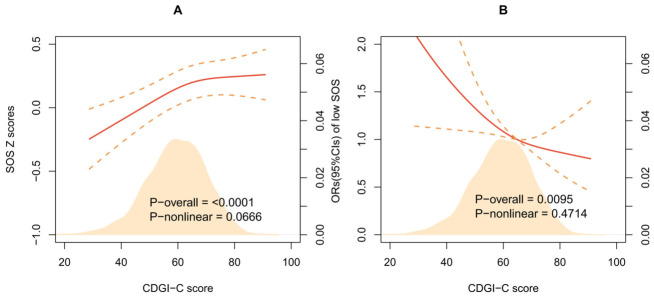
The association between CDGI-C and risks of low SOS. The restricted cubic splines (RCSs) with three knots of the dose–response relationship between CDGI-C and SOS Z scores (**A**), OR of low SOS (**B**). The models were adjusted for age, gender, weight status, income, parental education level, smoking, drinking status, MVPA, energy, stage of puberty, vitamin D supplement and calcium supplement.

After studying the dose–response relationship and several models between CDGI-C and bone health (both SOS Z scores and the risk of low SOS), the results demonstrated significant associations between CDGI-C and bone health outcomes. The restricted cubic spline (RCS) model with three knots demonstrated a statistically significant association between CDGI-C and SOS Z scores (*p*-overall < 0.0001, *p*-nonlinear = 0.0666, Figure 2), as well as low SOS status (*p*-overall = 0.0095, *p*-nonlinear = 0.4714, Figure 2), showing a decrease in OR of low SOS status and an upward trend of SOS Z scores as dietary scores increased. A 10-point rise in the CDGI-C was associated with a 0.08 increase in the SOS Z score (95% CI: 0.04, 0.11, Table 3) according to the fully adjusted GLM. Compared to Q1, SOS Z scores were significantly elevated in Q2, Q3, and Q4, with β values increasing from 0.12 (95% CI: 0.01, 0.22) to 0.20 (95% CI: 0.09, 0.31) and then 0.18 (95% CI: 0.08, 0.29, *p* for trend < 0.001). Furthermore, the fully adjusted binary logistic regression model reveals that each 10-point increase in the CDGI-C scores reduced the risk of low SOS by 15% (OR: 0.85, 95% CI: 0.76, 0.94, Table 4). Compared with participants of the lowest quartiles of CDGI-C, the reduction in low SOS risk progressed for successive quartiles, ranging from 8% (OR: 0.91, 95% CI: 0.67, 1.26) to 22% (OR: 0.78, 95% CI: 0.56, 1.09) and then 29% (OR: 0.71, 95% CI: 0.50, 1.00, *p* for trend = 0.032), indicating an association between higher CDGI-C scores and lower risk of low SOS.

### 3.3. Association Between 14 Dietary Items in the CDGI-C and Bone Health

From the generalized linear models and binary logistic regression models between 14 dietary items in the CDGI-C and bone health (both SOS Z scores and the risk of low SOS), the results demonstrated differential associations between individual dietary components and SOS. Model 1 (unadjusted model) showed that the dark green vegetables, fruits, dairy and dairy products, nuts, and seafood were all significantly positively associated with SOS Z scores (*p*-value < 0.05, Table 5). Among these, dark green vegetables and dairy and dairy products were reported to have the most significant associations, and the corresponding β values were 0.03 (95% CI: 0.01, 0.06) and 0.03 (95% CI: 0.01, 0.04). After adjusting for sociodemographic, lifestyle, pubertal, and supplementation variables (Model 4), dark green vegetables, dairy and dairy products, seafood and fish remained positively associated with SOS Z scores, and the corresponding β values were 0.03 (95% CI: 0.01, 0.05), 0.02 (95% CI: 0.01, 0.04), 0.02 (95% CI: 0.00, 0.03) and 0.01 (95% CI: 0.00, 0.02). However, only dairy and dairy products and seafood remained positive after Benjamini–Hochberg false discovery rate (FDR) correction. In Model 5, adjusting for another 13 dietary items, dark green vegetables, dairy and dairy products, and seafood remained significantly positively associated with SOS Z scores, and the corresponding β values were 0.03 (95% CI: 0.01, 0.05), 0.02 (95% CI: 0.00, 0.03) and 0.01 (95% CI: 0.00, 0.03), but no dietary item reached statistical significance after FDR. Similarly, results from the binary logistic regression model revealed that higher scores for grain and legumes, dark green vegetables, fruits, dairy and dairy products, beans, nuts, and seafood were significantly associated with a reduced risk of low SOS (*p*-value < 0.05, Table S2) in Model 1 (unadjusted model). However, no dietary item was found to have a statistically significant association with low SOS risk after adjusting for age, gender, body weight status, income, parental education level, energy intake, MVPA, stage of puberty, smoking status, drinking status, vitamin D and calcium supplementation, and 13 other dietary items. Meanwhile, dark green vegetables, dairy and dairy products, and nuts continued to be associated with a decreased risk of SOS in Model 4 (unadjusted for another 13 dietary items), still without statistical significance after FDR.

### 3.4. Stratified Analyses of the Association Between CDGI-C and Bone Health

**Figure 3:**
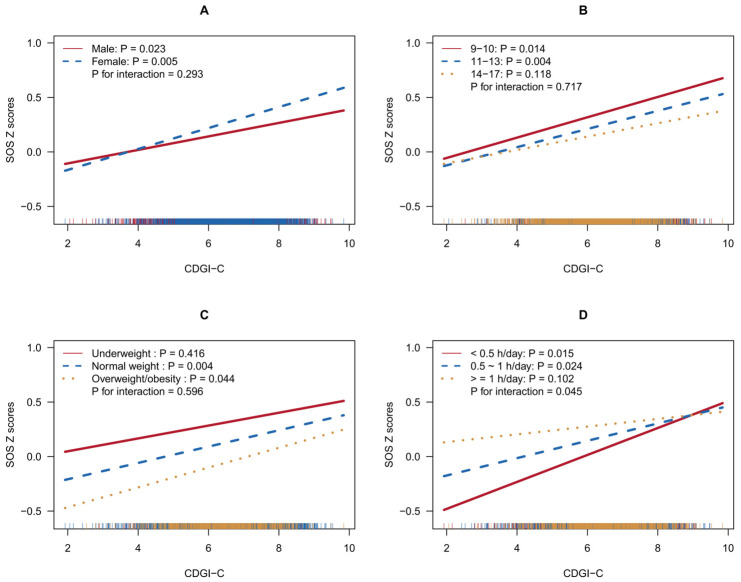
The association between serum CDGI-C and SOS was stratified by age (**A**), gender (**B**), weight status (**C**), and stage of MVPA (**D**). Visualization of interactions based on generalized linear models. The models were adjusted for age, gender, weight status, income, parental education level, smoking, drinking status, MVPA, energy, stage of puberty, vitamin D supplement and calcium supplement. Analyses were stratified by gender (male or female), age (9–10, 11–13 or 14–17 years), stage of MVPA (<0.5, 0.5~1, ≥1 h per day), and weight status (underweight, normal, overweight or obese).

We conducted stratified analyses, and the results showed that the association between CDGI-C and bone health (SOS Z scores) was not statistically significantly modified by gender (male or female), age (9–10 years, 11–13 years or 14–17 years), or weight status (underweight, normal weight, overweight, or obesity) (*p* for interaction > 0.05 for both, Table S3, Figure 3). However, we observed an interaction between CDGI-C and participants’ MVPA (<0.5 h/day, 0.5–1 h/day or ≥1 h/day) (*p* for interaction = 0.045). The positive association between CDGI-C and SOS Z scores gradually weakened as MVPA levels increased. Specifically, the strongest association was observed among the adolescents with insufficient activity (<0.5 h/day), while the effect weakened in the moderate activity group (0.5–1 h/day) and was no longer significant in the sufficient activity group (≥1 h/day), with the β values ranging from 0.13 (95% CI: 0.06, 0.21) to 0.07 (95% CI: 0.02, 0.12), and then 0.04 (95% CI: −0.02, 0.10).

## 4. Discussion

Based on this cross-sectional survey of 3299 Chinese children and adolescents aged 9–17 years, we examined the association between dietary quality assessed by CDGI-C and bone health measured by SOS. Results revealed that higher CDGI-C scores correlate with greater SOS values. Participants with higher CDGI-C had significantly higher SOS than those in the lowest quartile, indicating an association between superior nutritional quality and denser bone structure. Furthermore, we found that dairy and dairy products and seafood are key dietary components positively associated with SOS.

To our knowledge, this study is the first to focus on the association between dietary quality assessed by CDGI-C and bone health by QUS measurements in individuals, revealing a significant independent association between the two. It is in line with the impacts of bone health as determined by alternative devices such as DXA and dietary quality as assessed by other dietary indexes. For example, four scoring systems—the Healthy Eating Index-2005 (HEI-2005, 12 items), the alternate Healthy Eating Index (aHEI, eight items), the Diet Quality Index-International (DQI-I, 17 items), and the alternate Mediterranean Diet Score (aMed, nine items)—were used in a case–control study [32] among elderly urban Chinese in Guangdong, China, to assess dietary quality and look into its relationship to hip fracture risk. It was found that higher dietary index scores were correlated with higher bone mineral density (BMD). A case–control study in Iran [16] similarly assessed dietary quality using HEI and DQI to examine the relationship between dietary quality and BMD in postmenopausal Iranian women with osteoporosis. Results indicated that higher dietary quality scores were positively associated with greater femoral and lumbar BMD, suggesting that higher dietary quality is associated with better bone health in this population. However, previous studies on overall dietary quality have primarily focused on adults or the elderly, leaving a gap in research exploring the relationship between dietary quality and bone health in children and adolescents. This can be the result of difficulties in gathering and precisely quantifying dietary information for this population. Therefore, the relationship between dietary quality and bone health in children and adolescents requires further investigation across broader populations.

According to a Chinese study [18], which used DXA to measure left forearm BMD in individuals ages 9 to 12, adhering to the CDGI-C dietary pattern, maintaining a plant-animal balanced pattern, and moderate adherence to the bean–dairy pattern were positively associated with better bone health and a lower risk of low BMD. Though it further supported our findings that children’s bone health is positively associated with following a reasonable dietary structure, the study’s CDGI-C scoring range (0–75 points) fell below the predetermined scoring criteria because of lacking information on dark green vegetables, salt, oil, and sugar intake. Another study [9] examining the associations between dietary quality and areal bone mineral density in youth aged 10 to 23 years with healthy weight, obesity, and type 2 diabetes (T2D) discovered that higher HEI scores were correlated with higher BMD Z scores (*p* = 0.025), indicating a positive association between dietary quality and bone density, which is in line with our findings.

Our study’s results are broadly in line with previous research based on dietary patterns or dietary quality scores and bone health but further complement the evidence for Chinese children and adolescents by more precisely employing the CDGI-C as an evaluation tool, which aligns with the Dietary Guidelines for Chinese Residents (2016). Dairy and dairy products and seafood showed relatively stable positive correlations with bone health (SOS Z scores) at the dietary item level. Regarding potential mechanisms, higher dietary quality frequently entails richer micronutrient intake, lower inflammation [33], and more balanced energy allocation. Dairy products are key sources of dietary calcium and high-quality protein. Studies have shown that dairy products exert a dual regulatory effect on bone metabolism [34]. Milk basic protein promotes osteoblast proliferation and differentiation by directly stimulating gastric mucosal secretion of ghrelin [35]. Meanwhile, various bioactive components in dairy products—including lactoperoxidase, osteoprotegerin, and extracellular vesicles—inhibit osteoclast formation and bone resorption through mechanisms such as upregulating the OPG/RANKL ratio, suppressing RANKL signaling, and downregulating the expression of the osteoclast transcription factors c-Fos and NFATc1 [36,37,38,39]. Seafood is a rich source of omega-3 and vitamin D. Vitamin D regulates calcium metabolism [40], while omega-3 fatty acids promote bone health by inhibiting osteoclast differentiation and activity and increasing osteoblast production and mineralization [41]. These findings suggest that, in addition to improvements in the overall quality of diet, food categories rich in calcium, high-quality protein, and various micronutrients may be important for bone mass accumulation during childhood and adolescence. Furthermore, our results show that the ways in which certain dietary categories contribute to lowering low SOS risk are not mutually exclusive. This implies that, to reduce the occurrence of decreased BMD in children and adolescents, it is essential to focus on the synergistic effect of specific food groups (e.g., vegetables, dairy, and seafood) within an overall healthy dietary pattern.

Based on these findings, we propose some practical dietary considerations. First, children and adolescents could be encouraged to follow a balanced and diverse dietary pattern that includes higher intakes of foods such as vegetables, fruits, dairy products, legumes, nuts, and seafood, while limiting oil, salt, and sugary drinks. Second, among specific food choices, particular attention may be given to dairy products and seafood. Dairy products are rich in calcium and high-quality protein, while seafood provides high-quality protein and vitamin D. Both food groups showed the most robust positive associations with bone health in this study. However, we note that overall dietary quality (assessed by the Chinese Dietary Guidelines Compliance Score) had a stronger effect size than any individual food component. Therefore, while dairy products and seafood appear to be valuable components, the priority could be to improve overall dietary quality. In summary, adopting a healthy dietary pattern characterized by overall dietary quality—with dairy products and seafood as key contributory components—may be associated with better bone health in children and adolescents.

However, several potentially important factors related to bone metabolism, including sunlight exposure, pubertal hormones, and specific bone turnover biomarkers, were not assessed in the present study. These factors may influence bone health directly or indirectly and could therefore contribute to residual confounding. For example, sunlight exposure affects endogenous vitamin D synthesis, while pubertal hormonal changes play a critical role in bone accrual during adolescence. Furthermore, bone turnover biomarkers may reflect dynamic processes of bone formation and resorption that were not captured by QUS measurements alone. If these unmeasured factors are positively associated with both dietary quality and bone health, the observed associations may be overestimated; conversely, if the associations are inconsistent, the direction of confounding is more difficult to predict. Although we adjusted for several relevant covariates, including age, sex, BMI, and MVPA, the potential influence of these unmeasured or unanalyzed factors cannot be completely excluded.

Additionally, we conducted stratified analyses to examine the relationship between CDGI-C and bone health, accounting for factors such as gender, age, MVPA levels, and body weight status. Gender, age, and body weight status did not significantly influence the association, whereas MVPA levels did. The association between CDGI-C and bone health was most pronounced among adolescents with insufficient physical activity (<0.5 h/day). This suggests that the association between dietary quality and bone health may be more pronounced when physical activity is insufficient. One possible explanation is that adequate physical activity itself provides substantial osteogenic stimulation through mechanical loading, which plays an important role in bone formation and remodeling [42]. This effect may partially compensate for differences in dietary quality among more physically active adolescents. In addition, adolescents with higher MVPA levels may also engage in other healthier lifestyle behaviors that support bone health, thereby reducing the relative contribution of dietary quality alone. In contrast, among adolescents with insufficient physical activity, bone health may be more sensitive to nutritional status, making dietary quality more strongly associated with SOS. A study examining the effects of physical activity and diet on BMD in children and adolescents [43] demonstrated that sufficient physical activity may positively influence bone mass accumulation and BMD enhancement. Therefore, our findings suggest that both balanced nutrition and adequate physical activity are important for pediatric bone health, while the relative contribution of dietary quality may be more apparent in individuals with lower activity levels.

The primary strengths of this study lie in its use of CDGI-C for dietary quality assessment and its exploration of the association between CDGI-C and bone health. Other specific advantages include the following: first, the study employed a multistage stratified cluster random sampling method, yielding a representative sample covering different age groups. This approach enables reliable secondary analysis and ensures sufficient statistical power. Second, the study encompassed all dietary indicators within the CDGI-C scoring system, adhering to the predetermined CDGI-C scoring criteria to accurately assess dietary quality among children and adolescents. Third, we employed QUS to evaluate bone health status, which offers greater cost-effectiveness compared to DXA [23]. Fourth, our study is the first to investigate the association between CDGI-C and bone health measured by QUS, while also examining the independent roles of 14 specific dietary items. This provides valuable insights into the association between dietary quality and bone health in children and adolescents.

Importantly, although the observed effect sizes were relatively modest, the findings may still have important clinical and public health implications. Bone development during childhood and adolescence is a long-term cumulative process, and even small improvements in bone health indicators during these critical growth periods may contribute to achieving higher peak bone mass and lowering the future risk of osteoporosis and fractures in adulthood [44]. Moreover, dietary quality is a modifiable exposure at the population level. In this context, the observed 15% reduction in the risk of low SOS associated with every 10-point increase in CDGI-C score may represent a meaningful public health benefit, particularly given the high prevalence of suboptimal dietary behaviors among children and adolescents. Furthermore, compared with pharmacological interventions, the effects of overall dietary patterns on health outcomes are typically moderate because dietary exposures are multifactorial and influenced by complex behavioral and environmental determinants. Therefore, the present findings remain relevant for nutritional prevention strategies targeting pediatric bone health.

Although this study makes certain contributions, the following several limitations remain: first, as a cross-sectional survey, it cannot establish a causal relationship between dietary quality assessed by CDGI-C and bone health. To better understand diet‘s role in improving bone health and to confirm causal effects, future longitudinal research or dietary therapies are needed. Second, this study surveyed only children and adolescents in a specific Chinese city. The characteristics of this city may not be representative of the entire nation. Subsequent surveys should be conducted across multiple regions nationwide to support the findings of this study. Third, it should be noted that QUS does not measure areal bone mineral density directly as DXA does, and our results reflect bone properties rather than clinical diagnostic criteria for osteoporosis. Fourth, even though this study considered the influence of several confounding variables, there are still a lot of unknown factors remaining, which may also significantly impact the study and require continuous identification and adjustment to ensure the rigor of this research. Fifth, given the multiple comparisons performed across 14 dietary components, we applied the Benjamini–Hochberg FDR correction to reduce the risk of Type I error. However, no multiple comparison correction methods can completely eliminate the possibility of false-positive findings. Therefore, our FDR-corrected findings should be interpreted with caution, and replication in independent cohorts is warranted. Finally, formal reliability assessment of the CDGI (2021)-C has not yet been explicitly reported in the literature. Although the index has demonstrated construct and content validity [18,45,46,47], further methodological studies evaluating its reliability would help strengthen the evidence base for its broader application.

## 5. Conclusions

In conclusion, this study revealed a strong positive linear association between dietary quality assessed by CDGI-C and bone health by QUS measurements. In particular, dairy and dairy products and seafood were identified as key dietary components positively associated with SOS. These findings have implications for nutritional healthcare policy and practice. CDGI-C could be integrated into school-based health surveillance to identify children with poor dietary quality and higher bone health risk. In regions with inadequate intake of dairy products and seafood, increasing the availability of these foods in school meal programs may be considered. Though the study demonstrated the association between dietary patterns and bone health, randomized controlled trials are needed to evaluate the effectiveness of interventions aimed at improving dietary quality and increasing dairy and seafood intake on bone health outcomes. Multicenter studies across diverse regions and populations are also warranted to validate the generalizability of the present findings.

## Figures and Tables

**Table 1:** Baseline characteristics of 3299 participants according to CDGI-C quartile.

Variables	Total	Q1	Q2	Q3	Q4	*p*
*n*	3299	825	825	825	824	
Age group, *n* (%)						<0.001 *
9–10	696 (21.1)	142 (17.2)	160 (19.4)	153 (18.5)	241 (29.2)	
11–13	1216 (36.9)	262 (31.8)	300 (36.4)	326 (39.5)	328 (39.8)	
14–17	1387 (42.0)	421 (51.0)	365 (44.2)	346 (41.9)	255 (30.9)	
Gender, *n* (%)						0.009 *
Male	1837 (55.7)	496 (60.1)	463 (56.1)	449 (54.4)	429 (52.1)	
Female	1462 (44.3)	329 (39.9)	362 (43.9)	376 (45.6)	395 (47.9)	
Paternal education level, *n* (%)						0.028 *
≤9 years	605 (18.3)	180 (21.8)	147 (17.8)	154 (18.7)	124 (15.0)	
∼12 years	764 (23.2)	198 (24.0)	202 (24.5)	188 (22.8)	176 (21.4)	
∼15 years	941 (28.5)	216 (26.2)	247 (29.9)	226 (27.4)	252 (30.6)	
≥16 years	948 (28.7)	224 (27.2)	217 (26.3)	246 (29.8)	261 (31.7)	
Missing	41 (1.2)	7 (0.8)	12 (1.5)	11 (0.3)	11 (1.3)	
Maternal education level, *n* (%)						0.006 *
≤9 years	721 (21.9)	207 (25.1)	171 (20.7)	185 (22.4)	158 (19.2)	
∼12 years	921 (27.9)	252 (30.5)	232 (28.1)	223 (27.0)	214 (26.0)	
∼15 years	923 (28.0)	201 (24.4)	249 (30.2)	237 (28.7)	236 (28.6)	
≥16 years	714 (21.6)	158 (19.2)	167 (20.2)	177 (21.5)	212 (25.7)	
Missing	20 (0.6)	7 (0.8)	6 (0.7)	3 (0.4)	4 (0.5)	
Household income, *n* (%)						0.033 *
<120,000 CNY per year	935 (28.3)	264 (32.0)	229 (27.8)	231 (28.0)	211 (25.6)	
∼250,000 CNY per year	1126 (34.1)	264 (32.0)	288 (34.9)	296 (35.9)	278 (33.7)	
>=250,000 CNY per year	1214 (36.8)	289 (35.0)	307 (37.2)	289 (35.0)	329 (39.9)	
Missing	24 (0.7)	8 (1.0)	1 (0.1)	9 (1.1)	6 (0.7)	
Weight status, *n* (%)						0.595
Underweight	243 (7.4)	55 (6.7)	59 (7.2)	62 (7.5)	67 (8.1)	
Normal weight	2255 (68.4)	568 (68.8)	553 (67.0)	555 (67.3)	579 (70.3)	
Overweight	490 (14.9)	120 (14.5)	125 (15.2)	133 (16.1)	112 (13.6)	
Obesity	311 (9.4)	82 (9.9)	88 (10.7)	75 (9.1)	66 (8.0)	
Stage of puberty, *n* (%)						<0.001 *
Pre-puberty	343 (10.4)	70 (8.5)	85 (10.3)	77 (9.3)	111 (13.5)	
Mid-puberty	887 (26.9)	172 (20.8)	216 (26.2)	206 (25.0)	293 (35.6)	
Post-puberty	2069 (62.7)	583 (70.7)	524 63.5)	542 (65.7)	420 (51.0)	
MVPA, *n* (%)						<0.001 *
<0.5 h perd	770 (23.3)	240 (29.1)	208 (25.2)	184 (22.3)	138 (16.7)	
∼1 h perd	1505 (45.6)	357 (43.3)	365 (44.2)	395 (47.9)	388 (47.1)	
∼3 h perd	875 (26.5)	188 (22.8)	221 (26.8)	208 (25.2)	258 (31.3)	
>3 h perd	135 (4.1)	36 (4.4)	29 (3.5)	35 (4.2)	35 (4.2)	
Missing	14 (0.4)	4 (0.5)	2 (0.2)	3 (0.4)	5 (0.6)	
Smoking, *n* (%)						0.021 *
No	3215 (97.5)	792 (96.0)	810 (98.2)	803 (97.3)	810 (98.3)	
Yes	80 (2.4)	33 (4.0)	14 (1.7)	20 (2.4)	13 (1.6)	
Missing	4 (0.1)	0 (0.0)	1 (0.1)	2 (0.2)	1 (0.1)	
Drinking status, *n* (%)						<0.001 *
Never	2818 (85.4)	553 (67.0)	703 (85.2)	766 (92.8)	796 (96.6)	
<1 standard drink/month	369 (11.2)	202 (24.5)	98 (11.9)	43 (5.2)	26 (3.2)	
≥1 standard drink/month	111 (3.4)	70 (8.5)	24 (2.9)	15 (1.8)	2 (0.2)	
Missing	1 (0.0)	0 (0.0)	0 (0.0)	1 (0.1)	0 (0.0)	
Vitamin D supplement, *n* (%)						0.006 *
No	2841 (86.1)	718 (87.0)	733 (88.8)	712 (86.3)	678 (82.3)	
Yes	454 (13.8)	107 (13.0)	90 (10.9)	112 (13.6)	145 (17.6)	
Missing	4 (0.1)	0 (0.0)	2 (0.2)	1 (0.1)	1 (0.1)	
Calcium supplement, *n* (%)						0.504
No	2757 (83.6)	703 (85.2)	685 (83.0)	693 (84.0)	676 (82.0)	
Yes	540 (16.4)	122 (14.8)	139 (16.8)	131 (15.9)	148 (18.0)	
Missing	2 (0.1)	0 (0.0)	1 (0.1)	1 (0.1)	0 (0.0)	
SOS, m/s (median [IQR])	1551.00 [1533.00, 1576.00]	1549.00 [1530.00, 1572.00]	1550.00 [1533.00, 1576.00]	1554.00 [1533.00, 1579.00]	1554.00 [1535.00, 1575.00]	0.015 *
SOS Z_score (median [IQR])	0.17 [−0.45, 0.89]	0.03 [−0.57, 0.73]	0.12 [−0.47, 0.87]	0.21 [−0.45, 0.97]	0.24 [−0.33, 0.97]	<0.001 *
Energy, Kca/d (median [IQR])	2178.55 [1764.10, 2668.82]	2283.10 [1767.21, 2867.69]	2154.95 [1727.64, 2703.53]	2123.69 [1761.43, 2538.22]	2150.19 [1789.25, 2595.32]	<0.001 *

Abbreviation: CDGI-C, Chinese dietary guidelines index for Children and Adolescents; CNY, Chinese Yuan; MVPA, moderate to severe physical activity; SOS, speed of sound. Categorical variables were described as frequency (n) and percentage (%). For non-normally distributed variables tested by the Kolmogorov–Smirnov test, they were described as median and interquartile ranges [Median (IQR)]. * *p* < 0.05.

**Table 2:** The baseline scores of 14 dietary items in the CDGI-C according to CDGI-C quartile.

Variables	Total	Q1	Q2	Q3	Q4	*p*
Grain and legume scores	0.36 [0.02, 1.03]	0.12 [0.00, 0.59]	0.29 [0.00, 0.86]	0.43 [0.07, 1.07]	0.61 [0.21, 1.52]	<0.001
Vegetable scores	0.34 [0.15, 0.71]	0.24 [0.09, 0.56]	0.34 [0.16, 0.71]	0.36 [0.18, 0.72]	0.40 [0.20, 0.76]	<0.001
Dark green vegetables scores	5.00 [5.00, 5.00]	5.00 [0.77, 5.00]	5.00 [5.00, 5.00]	5.00 [5.00, 5.00]	5.00 [5.00, 5.00]	<0.001
Fruit scores	6.00 [2.64, 10.00]	3.30 [1.63, 6.15]	4.92 [2.29, 9.23]	6.15 [3.30, 10.00]	8.57 [6.00, 10.00]	<0.001
Dairy and dairy product scores	8.93 [5.95, 10.00]	7.39 [3.33, 10.00]	8.57 [5.48, 10.00]	9.32 [6.67, 10.00]	10.00 [8.33, 10.00]	<0.001
Bean scores	3.06 [1.01, 5.00]	1.88 [0.42, 5.00]	2.86 [0.89, 5.00]	3.11 [1.07, 5.00]	4.52 [2.26, 5.00]	<0.001
Nut scores	1.68 [0.00, 5.00]	0.00 [0.00, 2.63]	1.26 [0.00, 5.00]	1.89 [0.00, 5.00]	5.00 [1.26, 5.00]	<0.001
Seafood scores	2.97 [1.14, 6.28]	1.76 [0.53, 4.00]	2.41 [0.94, 5.50]	3.09 [1.33, 6.33]	4.88 [2.40, 8.78]	<0.001
Carbohydrate rate scores	5.00 [2.37, 5.00]	2.47 [2.17, 5.00]	5.00 [2.32, 5.00]	5.00 [2.45, 5.00]	5.00 [5.00, 5.00]	<0.001
Meat scores	0.00 [0.00, 5.18]	0.00 [0.00, 0.00]	0.00 [0.00, 2.67]	0.00 [0.00, 6.27]	3.74 [0.00, 7.69]	<0.001
Egg scores	3.85 [1.55, 8.70]	2.33 [0.31, 4.49]	3.16 [1.24, 7.35]	4.12 [2.33, 8.70]	8.43 [3.73, 9.56]	<0.001
Salt scores	10.00 [6.61, 10.00]	7.73 [2.47, 10.00]	10.00 [6.00, 10.00]	10.00 [8.03, 10.00]	10.00 [9.15, 10.00]	<0.001
Oil scores	10.00 [8.11, 10.00]	8.88 [4.51, 10.00]	10.00 [7.47, 10.00]	10.00 [9.45, 10.00]	10.00 [10.00, 10.00]	<0.001
Drink scores	10.00 [10.00, 10.00]	10.00 [0.00, 10.00]	10.00 [10.00, 10.00]	10.00 [10.00, 10.00]	10.00 [10.00, 10.00]	<0.001

Abbreviation: CDGI-C, Chinese dietary guidelines index for Children and Adolescents. Because 14 dietary items in the CDGI-C are non-normally distributed variables tested by the Kolmogorov–Smirnov test, they were described as median and interquartile ranges [Median (IQR)]. Q-values (FDR-adjusted *p*-values) were all <0.05 after Benjamini–Hochberg correction.

**Table 3:** Association between CDGI-C ^a^ and SOS Z scores ^b^.

	Model 1		Model 2		Model 3		Model 4	
	β (95% CI) ^c^	*p*-Value	β (95% CI) ^c^	*p*-Value	β (95% CI) ^c^	*p*-Value	β (95% CI) ^c^	*p*-Value
Q1	0 (Reference)		0 (Reference)		0 (Reference)		0 (Reference)	
Q2	0.09 (−0.01, 0.19)	0.091	0.09 (−0.02, 0.19)	0.104	0.12 (0.01, 0.22)	0.031	0.12 (0.01, 0.22)	0.031
Q3	0.17 (0.06, 0.27)	0.002	0.16 (0.05, 0.26)	0.003	0.20 (0.09, 0.30)	<0.001	0.20 (0.09, 0.31)	<0.001
Q4	0.18 (0.08, 0.29)	<0.001	0.15 (0.05, 0.26)	0.005	0.19 (0.08, 0.30)	0.001	0.18 (0.08, 0.29)	<0.001
*p* trend ^d^		<0.001		0.002		<0.001		<0.001
Per 10 score increment	0.07 (0.04, 0.10)	<0.001	0.06 (0.03, 0.10)	<0.001	0.08 (0.04, 0.11)	<0.001	0.08 (0.04, 0.11)	<0.001

Generalized linear models were used. Model 1 was the crude model with no adjustments. Model 2 was adjusted for age, gender and weight status. Model 3 was further adjusted for income, parental education level, smoking, drinking status, MVPA, energy and stage of puberty. Model 4 was further adjusted for vitamin D supplement and calcium supplement. ^a^ CDGI-C, Chinese dietary guidelines index for Children and Adolescents. ^b^ SOS, speed of sound. ^c^ β, estimates of regression coefficients; 95%CI, 95% confidence interval. ^d^ *p* value for trend was tested by using the median value with each quartile.

**Table 4:** Association between CDGI-C ^a^ and low SOS risk ^b^.

	Model 1		Model 2		Model 3		Model 4	
	OR (95%CI) ^c^	*p*-Value	OR (95%CI) ^c^	*p*-Value	OR (95%CI) ^c^	*p*-Value	OR (95%CI) ^c^	*p*-Value
Q1	0 (Reference)		0 (Reference)		0 (Reference)		0 (Reference)	
Q2	0.96 (0.71, 1.31)	0.816	0.96 (0.71, 1.31)	0.816	0.91 (0.66, 1.24)	0.543	0.92 (0.67, 1.26)	0.590
Q3	0.85 (0.62, 1.16)	0.301	0.85 (0.62, 1.17)	0.321	0.77 (0.56, 1.08)	0.130	0.78 (0.56, 1.09)	0.141
Q4	0.70 (0.51, 0.97)	0.034	0.75 (0.54, 1.05)	0.091	0.70 (0.49, 0.99)	0.046	0.71 (0.50, 1.00)	0.050
*p* trend ^d^		0.027		0.071		0.030		0.032
Per 10 score increment	0.87 (0.79, 0.96)	0.004	0.88 (0.80, 0.97)	0.011	0.84 (0.76, 0.94)	0.002	0.85 (0.76, 0.94)	0.003

Binary logistic models were used. Model 1 was the crude model with no adjustments. Model 2 was adjusted for age, gender and weight status. Model 3 was further adjusted for income, parental education level, smoking, drinking status, MVPA, energy and stage of puberty. Model 4 was further adjusted for vitamin D supplement and calcium supplement. ^a^ CDGI-C, Chinese dietary guidelines index for Children and Adolescents. ^b^ SOS, speed of sound. ^c^ OR, odds ratio; 95%CI, 95% confidence interval. ^d^ *p* value for trend was tested by using the median value with each quartile.

**Table 5:** Association between 14 dietary items in the CDGI-C ^a^ and SOS Z scores ^b^.

Variables	Model 1		Model 2		Model 3		Model 4		Model 5	
	β (95% CI) ^c^	*p*-Value	β (95% CI) ^c^	*p*-Value	β (95% CI) ^c^	*p*-Value	β (95% CI) ^c^	*p*-Value	β (95% CI) ^c^	*p*-Value
Grain and legume	0.03 (−0.00, 0.06)	0.077	0.03 (−0.01, 0.06)	0.126	0.02 (−0.02, 0.05)	0.283	0.02 (−0.02, 0.05)	0.309	0.01 (−0.02, 0.05)	0.517
Vegetables	−0.05 (−0.11, 0.02)	0.189	−0.05 (−0.11, 0.02)	0.186	−0.06 (−0.14, 0.01)	0.075	−0.06 (−0.13, 0.01)	0.083	−0.10 (−0.17, −0.02)	0.013
Dark green vegetables	0.03 (0.01, 0.06)	0.005 *	0.03 (0.00, 0.05)	0.018	0.03 (0.01, 0.05)	0.013	0.03 (0.01, 0.05)	0.011	0.03 (0.01, 0.05)	0.011
Fruits	0.02 (0.00, 0.03)	0.005 *	0.01 (0.00, 0.02)	0.036	0.01 (−0.00, 0.02)	0.120	0.01 (−0.00, 0.02)	0.123	0.01 (−0.01, 0.02)	0.436
Dairy and dairy products	0.03 (0.01, 0.04)	<0.001 *	0.03 (0.01, 0.04)	<0.001 *	0.02 (0.01, 0.04)	<0.001 *	0.02 (0.01, 0.04)	<0.001 *	0.02 (0.00, 0.03)	0.009
Beans	0.01 (−0.01, 0.03)	0.228	0.02 (−0.00, 0.03)	0.116	0.01 (−0.01, 0.03)	0.269	0.01 (−0.01, 0.03)	0.277	0.00 (−0.02, 0.02)	0.853
Nuts	0.02 (0.00, 0.04)	0.016	0.02 (0.00, 0.04)	0.037	0.01 (−0.00, 0.03)	0.129	0.01 (−0.00, 0.03)	0.139	0.01 (−0.01, 0.02)	0.562
Seafood	0.02 (0.01, 0.03)	0.001 *	0.02 (0.01, 0.03)	0.001 *	0.02 (0.00, 0.03)	0.006 *	0.02 (0.00, 0.03)	0.006 *	0.01 (0.00, 0.03)	0.025
Carbohydrate rate	−0.00 (−0.03, 0.03)	0.871	−0.01 (−0.03, 0.02)	0.641	0.00 (−0.03, 0.03)	0.971	−0.00 (−0.03, 0.03)	0.987	0.00 (−0.03, 0.03)	0.970
Meat	0.00 (−0.01, 0.01)	0.602	0.00 (−0.01, 0.01)	0.664	0.01 (−0.00, 0.02)	0.224	0.01 (−0.00, 0.02)	0.219	0.01 (−0.00, 0.02)	0.226
Egg	0.01 (−0.00, 0.02)	0.051	0.01 (0.00, 0.02)	0.021	0.01 (0.00, 0.02)	0.030	0.01 (0.00, 0.02)	0.029	0.01 (−0.00, 0.02)	0.130
Salt	0.00 (−0.01, 0.01)	0.872	−0.00 (−0.01, 0.01)	0.913	0.01 (−0.01, 0.02)	0.477	0.01 (−0.01, 0.02)	0.486	−0.03 (−0.07, 0.02)	0.238
Oil	0.00 (−0.01, 0.02)	0.469	0.00 (−0.01, 0.02)	0.761	0.01 (−0.01, 0.03)	0.237	0.01 (−0.01, 0.03)	0.247	0.04 (−0.01, 0.09)	0.118
Drink	−0.01 (−0.02, 0.01)	0.334	−0.01 (−0.02, 0.00)	0.074	−0.02 (−0.04, 0.00)	0.122	−0.02 (−0.04, 0.00)	0.111	−0.02 (−0.04, 0.00)	0.064

Generalized linear models were used. Model 1 was the crude model with no adjustments. Model 2 was adjusted for age, gender and weight status. Model 3 was further adjusted for income, parental education level, smoking, drinking status, MVPA, energy and stage of puberty. Model 4 was further adjusted for vitamin D supplement and calcium supplement. Model 5 was further adjusted for another 13 dietary items. ^a^ CDGI-C, Chinese dietary guidelines index for Children and Adolescents. ^b^ SOS, speed of sound. ^c^ β, estimates of regression coefficients; 95%CI, 95% confidence interval. * Q-values (FDR-adjusted *p*-values) were <0.05 after Benjamini–Hochberg correction.

## Data Availability

Owing to contractual obligations and privacy regulations, the dataset supporting this manuscript is not publicly accessible. The corresponding codebook and analytical codes can be obtained upon reasonable request at wyj@gzhmu.edu.cn.

## Supplementary Materials

The following supporting information can be downloaded at https://www.mdpi.com/article/10.3390/nu18111812/s1, Table S1: CDGI(2021)—C evaluation indicators; Table S2: Association between 14 dietary items in the CDGI-C and low SOS risk; Table S3: Stratified analyses of potential modification effect for the association between CDGI-C and SOS Z score.
