# Supplementary material for: Adherence to Chinese Dietary Guidelines Is Associated with Better Bone Status in School-Aged Children and Adolescents
Source: Nutrients. 2026 Jun 4;18(11):1812. doi: 10.3390/nu18111812 (PMC13258581; doi:10.3390/nu18111812)
Supplement: Supplementary file 1 [file nutrients-18-01812-s001.zip › nutrients-4298711-supplementary.pdf]

Adherence to Chinese Dietary Guidelines Is Associated With Better Bone Status in School-Aged Children and Adolescents

Supplementary online content

Table S1. CDGI(2021) - C evaluation indicators

Table S2. Association between 14 dietary items in the CDGI-C and low SOS risk

Table S3. Stratified analyses of potential modification effect for the association between CDGI-C and SOS Z score

Table S1. CDGI(2021)-C evaluation indicators [a].

	RIs [b]	MinS [c]	MaxS [d]	MA [e]
**9~10 years old**				
Carbohydrate rate	50%-65%	0% or 100%	50%-65%	5
Grain and legume	30-70 g/d	0 g/d	≥50 g/d	5
Vegetable	300 g/d	0 g/d	≥300 g/d	5
Dark green vegetables	≥1/2	0 g/d	≥1/2	5
Fruits	150~200 g/d	0 g/d	≥175 g/d	10
Dairy and dairy product	300 g/d	0 g/d	≥300 g/d	10
Beans	15 g/d	0 g/d	≥15 g/d	5
Nuts			≥0 g/d	5
Meat	40 g/d	0 g/d or ≥80 g/d	40 g/d	10
Egg	25-40 g/d	0 g/d or ≥65 g/d	25-40 g/d	10
Seafood	40 g/d	0 g/d	≥40 g/d	10
Salt	<4 g/d	≥8 g/d	<4 g/d	10
Oil	20-25 g/d	≥40 g/d	≤20 g/d	10
Drink	>0 g/d	>0 g/d	0 g/d	10
**11~13 years old**				
Carbohydrate rate	50%-65%	0% or 100%	50%-65%	5
Grain and legume	30-70 g/d	0 g/d	≥50 g/d	5
Vegetable	400-450 g/d	0 g/d	≥425 g/d	5
Dark green vegetables	≥1/2	0 g/d	≥1/2	5
Fruits	200-300 g/d	0 g/d	≥250 g/d	10
Dairy and dairy product	300 g/d	0 g/d	≥300 g/d	10
Beans	15 g/d	0 g/d	≥15 g/d	5
Nuts	7-10 g/d	0 g/d	≥8.5 g/d	5
Meat	50 g/d	0 g/d or ≥100 g/d	50 g/d	10
Egg	40-50 g/d	0 g/d or ≥90 g/d	40-50g/d	10
Seafood	50 g/d	0 g/d	≥50 g/d	10
Salt	<6 g/d	≥12 g/d	<6 g/d	10
Oil	25~30 g/d	≥50 g/d	<25 g/d	10
Drink	>0 g/d	>0 g/d	0 g/d	10
**14~17 years old**				
Carbohydrate rate	50%-65%	0% or 100%	50%-65%	5
Grain and legume	50-100 g/d	0 g/d	≥75 g/d	5
Vegetable	450-500 g/d	0 g/d	≥475 g/d	5
Dark green vegetables	≥1/2	0 g/d	≥1/2	5
Fruits	300-350 g/d	0 g/d	≥325 g/d	10
Dairy and dairy product	300 g/d	0 g/d	≥300 g/d	10

	**RIs [b]**	**MinS [c]**	**MaxS [d]**	**MA [e]**
Beans	15-25 g/d	0 g/d	≥20 g/d	5
Nuts	7-10 g/d	0 g/d	≥8.5 g/d	5
Meat	50-75 g/d	0 g/d or ≥125 g/d	50-75 g/d	10
Egg	50 g/d	0 g/d or ≥100 g/d	50 g/d	10
Seafood	50-75 g/d	0 g/d	≥62.5 g/d	10
Salt	<6 g/d	≥12 g/d	<6 g/d	10
Oil	25-30 g/d	≥50 g/d	<25 g/d	10
Drink	0 g/d	>0 g/d	0 g/d	10

[a] **Adequacy component**: Score = MA, if intake ≥ RIs; otherwise Score = MA×(intake / RIs) .

Moderation component: Score = MA, if intake = RIs; Score = MA×(intake / RIs), if intake < RIs; Score = MA - [MA×(intake - MaxS) / RIs], if intake > RIs.

Restriction component: Score = MA, if intake ≤ MaxS; Score = MA - [MA×(intake - MaxS) / RIs], if MaxS < intake ≤ 2 times MaxS; Score = 0, if intake > 2 times MaxS.

[b] RIs, Recommended intakes.

[c] MinS, Minimum standards.

[d] MaxS, Maximum standards.

[e] MA, Maximum assignment, as well as maximum score.

[e] MA, Maximum assignment, as well as maximum score.

Table S2. Association between 14 dietary items in the CDGI-C [a] and low SOS risk [b].

Variables	Model 1		Model 2		Model 3		Model 4		Model 5	
	OR (95% CI) [c]	*p*-Value	OR (95% CI) [c]	*p*-Value	OR (95% CI) [c]	*p*-Value	OR (95% CI) [c]	*p*-Value	OR (95% CI) [c]	*p*-Value
**Grain and legume**	0.89 (0.79, 0.99)	0.038	0.93 (0.82, 1.03)	0.186	0.94 (0.84, 1.06)	0.330	0.95 (0.84, 1.06)	0.370	0.97 (0.86, 1.09)	0.659
**Vegetables**	0.97 (0.78, 1.19)	0.765	1.03 (0.82, 1.27)	0.799	1.06 (0.84, 1.33)	0.602	1.05 (0.83, 1.32)	0.655	1.10 (0.86, 1.39)	0.413
**Dark green vegetables**	0.93 (0.88, 1.00)	0.033	0.94 (0.88, 1.00)	0.047	0.94 (0.88, 1.00)	0.052	0.94 (0.88, 1.00)	0.049	0.95 (0.88, 1.02)	0.118
**Fruits**	0.95 (0.92, 0.98)	0.003	0.97 (0.94, 1.01)	0.158	0.98 (0.94, 1.02)	0.398	0.98 (0.95, 1.02)	0.417	0.99 (0.95, 1.03)	0.639
**Dairy and dairy products**	0.94 (0.91, 0.97)	<0.001*	0.95 (0.91, 0.98)	0.004	0.95 (0.91, 0.99)	0.012	0.95 (0.92, 0.99)	0.014	0.96 (0.92, 1.00)	0.052
**Beans**	0.93 (0.88, 0.99)	0.016	0.94 (0.89, 1.00)	0.040	0.94 (0.89, 1.01)	0.072	0.95 (0.89, 1.01)	0.085	0.97 (0.91, 1.03)	0.278
**Nuts**	0.91 (0.86, 0.96)	<0.001*	0.92 (0.87, 0.97)	0.004	0.93 (0.88, 0.99)	0.015	0.93 (0.88, 0.99)	0.018	0.95 (0.89, 1.00)	0.074
**Seafood**	0.96 (0.93, 1.00)	0.043	0.98 (0.95, 1.02)	0.274	0.99 (0.95, 1.03)	0.506	0.99 (0.95, 1.02)	0.493	1.00 (0.96, 1.04)	0.860
**Carbohydrate rate**	1.03 (0.95, 1.13)	0.480	1.03 (0.94, 1.12)	0.520	1.02 (0.93, 1.12)	0.670	1.02 (0.93, 1.12)	0.644	1.04 (0.93, 1.15)	0.514
**Meat**	1.02 (0.98, 1.05)	0.300	0.99 (0.96, 1.02)	0.557	0.98 (0.94, 1.01)	0.248	0.98 (0.94, 1.01)	0.239	0.98 (0.94, 1.02)	0.333
**Egg**	0.98 (0.95, 1.02)	0.332	0.98 (0.95, 1.01)	0.280	0.99 (0.95, 1.02)	0.376	0.99 (0.95, 1.02)	0.394	0.99 (0.96, 1.03)	0.743
**Salt**	1.02 (0.99, 1.06)	0.232	1.00 (0.97, 1.04)	0.908	0.99 (0.94, 1.03)	0.578	0.99 (0.94, 1.03)	0.559	0.98 (0.83, 1.15)	0.771
**Oil**	1.02 (0.98, 1.06)	0.395	1.00 (0.96, 1.05)	0.892	0.99 (0.94, 1.04)	0.601	0.99 (0.94, 1.04)	0.594	1.01 (0.85, 1.21)	0.895
**Drink**	1.02 (0.98, 1.05)	0.315	1.03 (1.00, 1.07)	0.098	1.03 (0.96, 1.10)	0.448	1.03 (0.97, 1.10)	0.422	1.03 (0.97, 1.11)	0.382

Binary Logistic Models were used. Model 1 was the crude model with no adjustments. Model 2 was adjusted for age, gender and weight status. Model 3 was further adjusted for income, parental education level, smoking, drinking status, MVPA, energy and stage of puberty. Model 4 was further adjusted for vitamin D supplement and calcium supplement. Model 5 was further adjusted for another 13 dietary items.

[a] CDGI-C, Chinese dietary guidelines index for Children and Adolescents.

[b] SOS, speed of sound.

[c] OR, Odds Ratio; 95%CI, 95% confidence interval.

* Q-values (FDR-adjusted p-values) were < 0.05 after Benjamini-Hochberg correction.

Table S3. Stratified analyses of potential modification effect for the association between CDGI-C and SOS Z score [a].

	N	Q1	Q2	Q3	Q4	*P* trend [b]	Per 10 scores increment	*P* interaction [c]
**Gender**								
Male	1837	0 (Reference)	0.06 (-0.06, 0.19)	0.16 (0.03, 0.29)	0.13 (-0.00, 0.26)	0.023	0.06 (0.02, 0.10)	0.293
Female	1462	0 (Reference)	0.19 (0.00, 0.37)	0.25 (0.07, 0.44)	0.27 (0.08, 0.45)	0.005	0.10 (0.04, 0.16)	
**Age, years**								
9-10	696	0 (Reference)	0.04 (-0.20, 0.28)	0.28 (0.04, 0.53)	0.24 (0.01, 0.47)	0.014	0.10 (0.03, 0.17)	0.717
11-13	1216	0 (Reference)	0.16 (-0.01, 0.33)	0.24 (0.07, 0.41)	0.24 (0.07, 0.41)	0.004	0.09 (0.04, 0.14)	
14-17	1387	0 (Reference)	0.14 (-0.03, 0.30)	0.16 (-0.01, 0.33)	0.13 (-0.06, 0.32)	0.118	0.06 (0.01, 0.12)	
**Weight status**								
Underweight	243	0 (Reference)	0.08 (-0.31, 0.46)	0.10 (-0.27, 0.48)	0.16 (-0.23, 0.56)	0.416	0.07 (-0.06, 0.20)	0.596
Normal weight	2255	0 (Reference)	0.15 (0.02, 0.28)	0.19 (0.06, 0.32)	0.19 (0.06, 0.33)	0.004	0.08 (0.03, 0.12)	
Overweight/obesity	801	0 (Reference)	0.04 (-0.17, 0.25)	0.28 (0.06, 0.49)	0.16 (-0.06, 0.39)	0.044	0.08 (0.01, 0.15)	
**MVPA**								
<0.5 h/day	770	0 (Reference)	0.12 (-0.10, 0.34)	0.22 (-0.01, 0.44)	0.28 (0.03, 0.54)	0.015	0.13 (0.06, 0.21)	0.045
0.5-1h/day	1505	0 (Reference)	0.17 (0.01, 0.33)	0.17 (0.02, 0.33)	0.19 (0.03, 0.35)	0.024	0.07 (0.02, 0.12)	
>=1h/day	1010	0 (Reference)	0.08 (-0.12, 0.28)	0.22 (0.02, 0.43)	0.14 (-0.06, 0.34)	0.102	0.04 (-0.02, 0.10)	

[a] Generalized linear models were used. Adjusted by age, gender, weight status, income, parental education level, smoking, drinking status, MVPA, energy, stage of puberty, vitamin D supplement and calcium supplement, excluding the subgroup factor itself.

[b] *P* value for trend was test by using the median value with each quartile.

[c] *P* value for interaction was test by using log likelihood ratio test.
